# Comparative characteristics of homocysteine and uric acid in patients with Parkinson’s disease-related cognitive impairment and post-stroke cognitive impairment

**DOI:** 10.3389/fneur.2026.1819930

**Published:** 2026-04-24

**Authors:** Jinbang Zhan, Wenfeng Zhang, Shuhua Guan, Yanan Liu, Xiaofen Lin, Jinli Yang, Weihua Yuan, Longyu Zhou, Gan Huang

**Affiliations:** Yangjiang Hospital of Traditional Chinese Medicine, Affiliated to Guangzhou University of Chinese Medicine, Yangjiang, Guangdong, China

**Keywords:** cognitive impairment, homocysteine, MMSE, Parkinson’s disease, stroke, uric acid

## Abstract

**Objective:**

To compare homocysteine (Hcy) and uric acid (UA) levels between patients with Parkinson’s disease with cognitive impairment (PD-CI) and those with post-stroke cognitive impairment (PS-CI), and to analyze the correlation of these biomarkers with cognitive function and their heterogeneity across different disease types.

**Methods:**

Patients diagnosed with cognitive impairment who were admitted to the Department of Neurology at Yangjiang Hospital of Traditional Chinese Medicine between January 2025 and December 2025 were included (60 PD-CI; 60 PS-CI). Blood Hcy and UA levels were uniformly measured, and cognitive function was assessed using the Mini-Mental State Examination (MMSE). Covariance analysis compared biomarker level differences between groups. Spearman correlation analysis and multivariate linear regression models with interaction terms explored the relationship between biomarkers and MMSE scores. Binary logistic regression identified factors independently associated with disease type.

**Results:**

After adjusting for confounding factors, the PD-CI group exhibited significantly higher Hcy levels (adjusted mean difference: 2.55 μmol/L, 95% CI: 0.23–4.87, *p* = 0.032), while UA levels were significantly lower (adjusted mean difference: −34.66 μmol/L, 95% CI: −63.87 to −5.46, *p* = 0.020). In the overall sample, Hcy negatively correlated with MMSE (*r* = −0.309, *p* = 0.001). This association was numerically stronger in the PD-CI group (*r* = −0.456, *p* < 0.001) and weaker in the PS-CI group (*r* = −0.175, *p* = 0.180). However, the difference in correlation coefficients between the two groups did not reach statistical significance (*p* = 0.093). UA levels showed no significant linear or nonlinear association with MMSE scores in either group. Multivariate regression analysis confirmed that elevated Hcy levels maintained a persistent negative correlation with MMSE scores in both groups. Low educational attainment, absence of hypertension, and lower UA levels were identified as independently associated with PD-CI (compared to PS-CI).

**Conclusion:**

Patients with PD-CI and PS-CI exhibit distinct metabolic profiles, with the PD-CI group characterized by elevated Hcy and low UA levels. Although elevated Hcy is a common factor associated with cognitive impairment in both conditions, UA levels in this cohort were independently associated with PD-CI (relative to PS-CI), not with MMSE scores. These findings suggest potential differences in metabolic profiles and may offer new insights for clinical differential diagnosis. As an exploratory cross-sectional analysis, these findings require validation in prospective studies.

## 1 Background

Cognitive impairment is one of the major causes of disability in neurological diseases, with Parkinson’s disease (PD) with cognitive impairment (PD-CI) and post-stroke cognitive impairment (PS-CI) being the two most common types encountered in clinical practice. Although both manifest as cognitive impairment, their pathophysiological mechanisms differ fundamentally: PD-CI is characterized by abnormal accumulation of *α*-synuclein (α-syn) and degeneration of dopaminergic neurons, whereas PS-CI arises from focal or diffuse brain tissue damage caused by cerebrovascular events (1, 2). With the accelerating global aging population, the prevalence of cognitive impairment caused by neurodegenerative diseases and cerebrovascular diseases is rising significantly, imposing a heavy burden on public health systems (3). Therefore, identifying simple and accessible peripheral blood biomarkers not only deepens our understanding of disease mechanisms but also offers new targets for early clinical identification and intervention.

Homocysteine (Hcy), a sulfur-containing amino acid, exhibits metabolic disorders closely associated with the onset and progression of various neurological disorders. Research confirms that hyperhomocysteinemia contributes to neurodegenerative processes through multiple pathways, including oxidative stress, endothelial dysfunction, excitotoxicity, and DNA damage (4). In recent years, numerous clinical studies have demonstrated a significant association between elevated Hcy levels and cognitive decline in Parkinson’s disease patients, with similar trends observed in individuals with post-stroke cognitive impairment (5, 6). Uric acid (UA), the final product of purine metabolism in the human body, also serves as a crucial endogenous antioxidant, scavenging approximately 55% of extracellular free radicals (7). In recent years, the dual role of UA in neurological disorders has garnered significant attention. Basic research indicates that UA exerts neuroprotective effects by regulating oxidative stress and neuroinflammation; however, translating these findings into clinical interventions remains challenging (7). Epidemiological evidence reveals complex relationships between serum UA levels and neurological disorders as well as cognitive function: lower UA levels may increase the risk of neurodegenerative diseases, while both excessively high and low UA levels are associated with cerebrovascular events and poor outcomes (8, 9). This intricate association suggests that the relationship between UA and cognitive function may vary by disease type, necessitating refined analyses in specific populations.

Although the individual roles of Hcy and UA in PD-CI and PS-CI have been extensively studied, few investigations have directly compared the distribution characteristics of these two biomarkers across different types of cognitive impairment within the same cohort, nor examined their relationship patterns with cognitive function. PD-CI and PS-CI patients exhibit distinct pathological backgrounds and metabolic characteristics, suggesting Hcy and UA may play different roles in each condition. Elucidating these differences could enhance understanding of the unique mechanisms underlying cognitive impairment in both disorders and provide reference for clinical differential diagnosis and personalized interventions. Therefore, this single-center cross-sectional study enrolled 60 PD-CI and 60 PS-CI patients to systematically compare differences in Hcy and UA levels between groups, analyze their correlations with cognitive function, and investigate whether these relationships vary by disease type. This aims to provide new clinical evidence for metabolic biomarker research in these two common cognitive disorders.

## 2 Methods

### 2.1 Study population

This study is a single-center cross-sectional survey that consecutively enrolled 120 patients with cognitive impairment who visited the Department of Neurology at Yangjiang Hospital of Traditional Chinese Medicine from January 2025 to December 2025. Participants were stratified by etiology into the PD-CI group (*n* = 60) and PS-CI group (*n* = 60). Inclusion criteria were: (1) Age 40–85 years; (2) Mini-Mental State Examination (MMSE) scores stratified by educational attainment: illiterate ≤19 points, elementary school ≤22 points, junior high school and above ≤26 points (10); (3) Clear consciousness and ability to cooperate with scale assessments; (4) Voluntary participation and signed informed consent. Common exclusion criteria: (1) Severe dysfunction of major organs such as heart, liver, lungs, or kidneys; (2) Use of medications potentially affecting cognitive function within the past month; (3) Inability to complete neuropsychological assessment due to severe aphasia, visual/auditory impairment, or motor dysfunction; (4) Refusal to participate in the study. PD-CI group additionally requires: PD diagnosis per the 2016 Chinese Diagnostic Criteria for Parkinson’s Disease (11); PD-CI diagnosis per the 2012 International Movement Disorder Society Diagnostic Guidelines (12); exclusion of secondary Parkinsonian syndromes, Parkinson’s superimposed syndromes, and other causes of cognitive impairment. The PS-CI group must additionally meet the following criteria: Stroke diagnosis based on the 2023 Chinese Guidelines for the Diagnosis and Treatment of Acute Ischemic Stroke (13); PS-CI diagnosis referenced from the 2021 Expert Consensus on Management of Post-Stroke Cognitive Impairment (14); Requirement for a clearly defined stroke event with cognitive impairment occurring post-stroke and persisting for 3 to 6 months or longer; Exclusion of cognitive impairment not meeting the temporal relationship criteria or caused by other etiologies.

### 2.2 Clinical data collection and laboratory testing

We collected demographic information (age, sex, educational attainment), clinical history (hypertension, diabetes, disease duration), and laboratory indicators for all patients. Educational attainment was categorized as illiterate, elementary school, and junior high school or above. History of hypertension was defined as a prior diagnosis of hypertension with ongoing antihypertensive treatment; history of diabetes was defined as a prior diagnosis of diabetes with ongoing hypoglycemic therapy. Laboratory parameters were measured using fasting venous blood collected the morning after admission. Tests included estimated glomerular filtration rate [eGFR, CKD-EPI formula (15)], homocysteine (Hcy), uric acid (UA), mean corpuscular volume (MCV), red blood cell distribution width-standard deviation (RDW-SD), platelet count (PLT), mean platelet volume (MPV), neutrophil count (NEUT), lymphocyte count (LYMPH), and calculated neutrophil-to-lymphocyte ratio (NLR) and platelet-to-lymphocyte ratio (PLR). Cognitive function was assessed using the MMSE administered by uniformly trained neurologists within 24 h of enrollment.

### 2.3 Statistical analysis

Data analysis was performed using SPSS 27.0 software. Continuous variables meeting normal distribution were expressed as mean ± standard deviation (*x̄* ± *s*), and intergroup comparisons were conducted using independent samples t-tests. Continuous variables not meeting normal distribution were expressed as median (first quartile, third quartile) *M*(Q_1_, Q_3_), and intergroup comparisons were conducted using the Mann–Whitney *U* test. Categorical variables were expressed as frequency and percentage (*n*(%)), and intergroup comparisons were performed using the chi-square test. All tests were two-tailed, with *p* < 0.05 considered statistically significant. To control for confounding factors, analysis of covariance (ANCOVA) was used to compare adjusted differences in Hcy and UA levels between groups, with age, sex, education level, and eGFR as covariates (16–18). Results are presented as adjusted mean differences and their 95% confidence intervals (95% CI). This comparison was designated as the primary analysis; all other analyses were exploratory. Spearman’s rank correlation analysis assessed the relationship between Hcy, UA, and MMSE scores. Correlation coefficients (*r*) and their significance (*p*-values) were calculated for the overall sample, PD-CI group, and PS-CI group. Intergroup comparisons of correlation coefficients were tested using Fisher’s *Z*-transformation. To investigate the potential nonlinear (U-shaped) relationship between UA and MMSE scores, multivariate linear regression models were constructed within the PD-CI and PS-CI groups. MMSE scores served as the dependent variable, incorporating UA and its squared term (UA^2^). The significance of the UA^2^ term (*p* < 0.05) indicated a nonlinear association. To investigate whether the effects of Hcy and UA on cognitive function differ by disease type, two multivariate linear regression models were constructed with MMSE scores as the dependent variable: Model A included grouping variable (PD-CI = 1, PS-CI = 0), Hcy, and their interaction term (grouping × Hcy); Model B included grouping, UA, UA^2^, and their interaction terms (grouping × UA, grouping × UA^2^). Results were presented as unstandardized coefficient *B* (95% CI), standardized coefficient *β*, *t*-value, *p*-value, and model *R*^2^ and adjusted *R*^2^. Prior to linear regression analysis, UA and Hcy values were centered on group means or overall means to reduce multicollinearity. All models adjusted for age, sex, education level, and eGFR. Interaction analysis models additionally included variables associated with MMSE or grouping in univariate analysis with *p* < 0.05. Interactions were considered significant at *p* < 0.10. Binary logistic regression analysis was employed to further identify factors independently associated with disease type (PD-CI vs. PS-CI). Univariate analysis was first performed for each candidate variable, calculating crude OR values and 95% CI. Variables with *p* < 0.05 in univariate analysis were included in the multivariate model using the Enter method (forced entry method) along with baseline covariates specified in the protocol, yielding adjusted OR values and 95% CI. All regression models underwent multicollinearity diagnosis. Variate interdependence was assessed by calculating variance inflation factors (VIF), with VIF > 5 indicating multicollinearity. All tests were two-sided, with *p* < 0.05 considered statistically significant.

To address multiple testing for the primary outcomes, the Benjamini-Hochberg false discovery rate (FDR) correction was applied to the two comparisons in the primary analysis, with *q* < 0.05 considered significant. A sensitivity analysis was also performed by repeating the ANCOVA for the primary model with a reduced covariate set (age and sex only) to confirm the robustness of the findings.

### 2.4 Ethical approval

This research protocol has been approved by the Ethics Committee of Yangjiang Traditional Chinese Medicine Hospital (Ethics Approval Number: 20260227). All participants were fully informed of the study objectives and procedures and provided written informed consent. The research process strictly adheres to the Declaration of Helsinki and ethical guidelines for biomedical research involving human subjects.

## 3 Results

### 3.1 Baseline characteristics of the study population

Table 1 presents a comparison of baseline characteristics between the PD-CI and PS-CI groups. No significant differences were observed between the two groups in terms of age, disease duration, diabetes history, or most blood cell count parameters (MCV, RDW-SD, PLT, MPV, LYMPH) (*p* > 0.05). Regarding confounding factors requiring adjustment, the PD-CI group had a significantly lower proportion of males (*p* < 0.001), a significantly higher proportion of illiterate individuals (*p* < 0.001), a significantly lower proportion with a history of hypertension (*p* < 0.001), and significantly higher eGFR levels (*p* = 0.018). Inflammatory markers NEUT, NLR, and PLR were significantly lower in the PD-CI group than in the PS-CI group (*p* < 0.05). Regarding core indicators, Hcy levels showed no significant difference between groups (*p* = 0.128), while UA levels were significantly lower in the PD-CI group (*p* = 0.002). Variables exhibiting intergroup differences will be adjusted as covariates in subsequent regression analyses.

**Table 1:** Comparison of baseline characteristics between the two groups.

Variable	PD-CI group (*n* = 60)	PS-CI group (*n* = 60)	Statistic	*p* value
Age (years, *M*(Q_1_, Q_3_))	70.00 (63.00, 74.75)	68.00 (62.25, 73.75)	*Z* = −1.246	0.213
Sex (male, *n*(%))	16 (26.7)	40 (66.7)	χ^2^ = 19.286	<0.001
Education level (*n*(%))			χ^2^ = 25.387	<0.001
Illiterate	17 (28.3)	1 (1.7)		
Primary school	31 (51.7)	25 (41.7)		
Junior high school and above	12 (20.0)	34 (56.7)		
History of hypertension (*n*(%))	22 (36.7)	42 (70.0)	χ^2^ = 13.393	<0.001
History of diabetes (*n*(%))	10 (16.7)	15 (25.0)	χ^2^ = 1.263	0.261
Disease duration (years, *M*(Q_1_, Q_3_))	3.00 (2.00, 5.00)	3.00 (1.00, 5.00)	*Z* = −0.998	0.318
eGFR (mL/min/1.73m^2^, *M*(Q1, Q3))	104.50 (85.00, 121.00)	95.50 (69.25, 107.75)	*Z* = −2.360	0.018
Hcy (μmol/L, *M*(Q1, Q3))	12.30 (9.83, 15.40)	10.40 (8.65, 15.00)	*Z* = −1.522	0.128
UA (μmol/L, *x̄* ± s)	289.68 ± 72.79	335.36 ± 83.73	*t* = −3.189	0.002
MCV (fL, *M*(Q1, Q3))	89.05 (87.13, 91.68)	89.15 (86.33, 92.58)	*Z* = 0.013	0.990
RDW-SD (fL, *x̄* ± s)	42.31 ± 3.32	42.82 ± 2.45	*t* = −0.955	0.341
PLT (×10^9^/L, *M*(Q_1_, Q_3_))	224.50 (183.75, 255.50)	238.50 (202.00, 283.00)	*Z* = 1.464	0.143
MPV (fL, *M*(Q_1_, Q_3_))	8.40 (8.20, 9.08)	8.50 (8.10, 9.28)	*Z* = 0.371	0.711
NEUT (×10^9^/L, *M*(Q_1_, Q_3_))	3.95 (3.32, 4.93)	4.48 (3.57, 5.55)	*Z* = 2.113	0.035
LYMPH (×10^9^/L, *x̄* ± s)	2.00 ± 0.59	1.85 ± 0.67	*t* = 1.311	0.193
NLR (*M*(Q_1_, Q_3_))	1.98 (1.40, 2.89)	2.52 (1.71, 3.44)	*Z* = 2.441	0.015
PLR (*M*(Q_1_, Q_3_))	109.90 (90.21, 144.02)	127.17 (100.45, 183.02)	*Z* = 2.000	0.046
MMSE score (M(Q_1_, Q_3_))	18.00 (14.00, 21.00)	19.00 (17.00, 22.00)	*Z* = 1.245	0.213

PD-CI, Parkinson’s disease with cognitive impairment; PS-CI, post-stroke cognitive impairment; M(Q_1_, Q_3_), median (first quartile, third quartile); x̄ ± s, mean ± standard deviation; eGFR, estimated glomerular filtration rate; Hcy, homocysteine; UA, uric acid; MCV, mean corpuscular volume; RDW-SD, red blood cell distribution width-standard deviation; PLT, platelet count; MPV, mean platelet volume; NEUT, neutrophil count; LYMPH, lymphocyte count; NLR, neutrophil-to-lymphocyte ratio; PLR, platelet-to-lymphocyte ratio; MMSE, Mini-Mental State Examination. Statistics: t is the t value of independent samples *t*-test, Z is the standardized statistic of Mann–Whitney U test, χ^2^ is the chi-square value.

### 3.2 Comparison of Hcy and UA levels between the two groups

Table 2 presents the crude analysis and adjusted differences in Hcy and UA levels between the two patient groups. Crude analysis showed no significant difference in Hcy levels between the PD-CI group and the PS-CI group (*p* = 0.128); However, after adjusting for age, sex, education level, and eGFR, the PD-CI group exhibited significantly higher Hcy levels than the PS-CI group, with an adjusted mean difference of 2.55 (95% CI 0.23–4.87) μmol/L (*p* = 0.032). UA levels were significantly lower in the PD-CI group than in the PS-CI group in the crude analysis (*p* = 0.002), and the difference remained significant after adjustment, with an adjusted mean difference of −34.66 (95% CI -63.87 to −5.46) μmol/L (*p* = 0.020). These findings indicate that after adjusting for confounding factors, PD-CI patients exhibit higher Hcy levels and lower UA levels. This suggests that these two metabolic markers may be involved in the pathophysiological processes of the two types of cognitive impairment, albeit with differing effects.

**Table 2:** Comparison of Hcy and UA levels between the two groups.

Variable	PD-CI group (*n* = 60)	PS-CI group (*n* = 60)	Unadjusted analysis		Adjusted analysis	
			Statistic	*P* value	Adjusted mean difference (95% CI)	*P* value
Hcy (μmol/L)	12.30 (9.83, 15.40)	10.40 (8.65, 15.00)	Z = −1.522	0.128	2.55 (0.23, 4.87)	0.032
UA (μmol/L)	289.68 ± 72.79	335.36 ± 83.73	*t* = −3.189	0.002	−34.66 (−63.87, −5.46)	0.020

ANCOVA adjusted for age, sex, education level, and eGFR. Hcy presented as M (Q_1_, Q_3_). UA presented as x̄ ± s. After Benjamini-Hochberg FDR correction (*q* = 0.05), the adjusted between-group differences for both Hcy and UA remained significant (*q* = 0.040 for both).

### 3.3 Correlation analysis of Hcy and UA with cognitive function

Table 3 presents the Spearman correlation analysis results between Hcy, UA, and MMSE scores. In the overall sample, Hcy showed a significant negative correlation with MMSE (*r* = −0.309, *p* = 0.001), while UA demonstrated no significant correlation with MMSE (*p* = 0.642). Subgroup analysis revealed a significant moderate-strength negative correlation between Hcy and MMSE in the PD-CI group (*r* = −0.456, *p* < 0.001), whereas the correlation in the PS-CI group did not reach statistical significance (*r* = −0.175, *p* = 0.180). The comparison of correlation coefficients between the two groups did not reach statistical significance (*p* = 0.093), suggesting that the observed numerical difference may be due to chance and should be interpreted cautiously. UA showed no significant correlation with MMSE in either the PD-CI group (*r* = −0.196, *p* = 0.133) or the PS-CI group (*r* = 0.030, *p* = 0.822), with no statistically significant difference between groups (*p* = 0.222). These findings indicate that elevated Hcy levels correlate with cognitive decline, with this association reaching statistical significance only in the PD-CI group in this sample. However, no significant association between UA and cognitive function was observed in the study population.

**Table 3:** Correlation of Hcy and UA with MMSE (overall and by group).

Variable	Overall (*N* = 120)	PD-CI group (*n* = 60)	PS-CI group (*n* = 60)	*P* value for comparison of correlation coefficients
	*r* (*P*)	*r* (*P*)	*r* (*P*)	
Hcy	−0.309 (0.001)	−0.456 (<0.001)	−0.175 (0.180)	0.093
UA	−0.043 (0.642)	−0.196 (0.133)	0.030 (0.822)	0.222

r is the Spearman correlation coefficient, ranging from −1 to + 1; a positive value indicates a positive correlation, a negative value indicates a negative correlation, and the larger the absolute value, the stronger the correlation. The Fisher Z transformation was used to test the difference in correlation coefficients between the two groups.

### 3.4 Nonlinear relationship analysis between UA and cognitive function

Table 4 analyzed whether a nonlinear (U-shaped) relationship existed between UA and MMSE scores, tested separately in the PD-CI and PS-CI groups. Results showed that the squared term of UA (UA^2^) did not reach statistical significance in either the PD-CI group or the PS-CI group (PD-CI group *p* = 0.802, PS-CI group *p* = 0.936), indicating no U-shaped relationship between UA and cognitive function in either group. Simultaneously, the linear term of UA was also not statistically significant in either group (*p* > 0.05), consistent with the results of the correlation analysis in Table 3. Therefore, in the study population, neither a significant linear correlation nor a clear nonlinear relationship was found between UA and MMSE scores.

**Table 4:** Nonlinear relationship between UA and MMSE: quadratic regression analysis by group.

Group	Variable	Unstandardized coefficient B (95% CI)	Standardized coefficient β	*t* value	*P* value
PD-CI group	UA	−0.011 (−0.030, 0.008)	−0.152	−1.142	0.259
	UA^2^	−1.97 × 10^−5^ (−1.70 × 10^−4^, 1.20 × 10^−4^)	−0.029	−0.253	0.802
PS-CI group	UA	0.005 (−0.011, 0.021)	0.095	0.663	0.510
	UA^2^	−5.97 × 10^−6^ (−1.50 × 10^−4^, 1.38 × 10^−4^)	−0.011	−0.081	0.936

The B value for UA^2^ is expressed in scientific notation, e.g., −1.97 × 10^−5^ equals −0.0000197. Each model was adjusted for age, sex, education level, and eGFR. The standardized coefficient β was calculated based on the standard deviations of the independent variable and the dependent variable. UA was centered by the within-group mean before analysis. The variance inflation factor (VIF) for all variables in both models was less than 5.

### 3.5 Intergroup heterogeneity analysis of the relationship between Hcy, UA, and cognitive function

Table 5 presents the results of a multiple linear regression analysis with MMSE as the dependent variable, aiming to examine whether the relationship between Hcy and UA with cognitive function differs by disease type (PD-CI vs. PS-CI). Model A (Hcy interaction) revealed that Hcy was an independent negative predictor of MMSE (*B* = −0.207, 95% CI − 0.375 to −0.039; *β* = −0.263, *p* = 0.016), while the interaction term between grouping and Hcy was not statistically significant (*p* = 0.841), indicating that the negative impact of Hcy on cognitive function was consistent across both PD-CI and PS-CI groups. Model B (nonlinear interaction of UA) showed that the linear term of UA (*p* = 0.175), the quadratic term (*p* = 0.785), and their interactions with grouping (group × UA *p* = 0.696, group × UA^2^ *p* = 0.619) were all statistically insignificant. This suggests that no significant linear or nonlinear relationship exists between UA and MMSE scores, and that this relationship does not vary by disease type. The goodness-of-fit for the two models was *R*^2^ = 0.410 (adjusted *R*^2^ = 0.338) for Model A and *R*^2^ = 0.370 (adjusted *R*^2^ = 0.286) for Model B, indicating that the models explained approximately 41 and 37% of the variance in MMSE scores, respectively. These findings suggest that the negative impact of Hcy on cognitive function is consistent across both types of cognitive impairment. However, no significant association between UA and cognitive function was observed in the study population, nor were any intergroup differences identified.

**Table 5:** Multiple linear regression analysis: testing the interaction effects of Hcy and UA between groups with MMSE as the dependent variable.

Variable	Model A (Hcy)			Model B (UA)		
	*B* (95%CI)	β	*P* value	B (95%CI)	β	*P* value
Constant	29.778 (20.039, 39.517)	—	<0.001	33.602 (23.683, 43.521)	—	<0.001
Group (PD-CI)	−1.013 (−2.898, 0.873)	—	0.289	−0.061 (−2.189, 2.310)	—	0.957
Hcy	−0.207 (−0.375, −0.039)	−0.263	0.016	—	—	—
Group × Hcy	0.026 (−0.231, 0.283)	—	0.841	—	—	—
UA	−0.006 (−0.018, 0.006)	−0.096	0.305	−0.011 (−0.028, 0.005)	−0.177	0.175
UA^2^	—	—	—	1.991 × 10^−5^ (−1.23 × 10^−4^, 1.62 × 10^−4^)	0.032	0.785
Group × UA	—	—	—	0.004 (−0.016, 0.024)	—	0.696
Group × UA^2^	—	—	—	−5.169 × 10^−5^ (−2.55 × 10^−4^, 1.52 × 10^−4^)	—	0.619
Age	−0.071 (−0.179, 0.038)	−0.111	0.199	−0.117 (−0.225, −0.009)	−0.183	0.034
Sex (male)	0.716 (−1.100, 2.532)	—	0.436	−0.039 (−1.851, 1.773)	—	0.966
Education (illiterate)	−6.899 (−9.592, −4.206)	—	<0.001	−7.488 (−10.257, −4.718)	—	<0.001
Education (primary school)	−2.674 (−4.378, −0.970)	—	0.002	−2.748 (−4.541, −0.954)	—	0.003
eGFR	−0.015 (−0.046, 0.015)	−0.094	0.318	−0.013 (−0.044, 0.018)	−0.081	0.411
History of hypertension (no)	0.063 (−1.540, 1.666)	—	0.938	0.337 (−1.360, 2.035)	—	0.694
NEUT	0.041 (−0.676, 0.758)	0.011	0.911	−0.155 (−0.927, 0.617)	−0.043	0.691
NLR	−0.403 (−1.068, 0.261)	−0.150	0.232	−0.368 (−1.095, 0.359)	−0.137	0.318
PLR	−0.015 (−0.032, 0.002)	−0.185	0.085	−0.017 (−0.035, 0.001)	−0.210	0.071
Model *R*^2^/Adjusted *R*^2^	0.410/0.338	—	—	0.370/0.286	—	—

Group (PD-CI) with PS-CI group as reference; Sex (male) with female as reference; Education level with “junior high school and above” as reference; History of hypertension (no) with “yes” as reference. Standardized coefficients β were calculated only for continuous variables; β values for categorical variables are left blank. Hcy values were centered on the overall sample mean, and UA values were centered on group means before analysis. VIF for all variables in both models were <5.

### 3.6 Factors independently associated with disease type

Table 6 presents the results of binary logistic regression analysis to identify factors independently associated with disease type (PD-CI vs. PS-CI). Univariate analysis revealed that compared to the PS-CI group, patients in the PD-CI group exhibited the following characteristics: a lower proportion of males (*OR* = 0.182, *p* < 0.001), a higher proportion of illiterate (*OR* = 48.167, *p* < 0.001) and primary school education (*OR* = 3.513, *p* = 0.003), a lower proportion with a history of hypertension (*OR* = 4.030, *p* < 0.001), higher eGFR levels (*OR* = 1.012, *p* = 0.054), lower UA levels (*OR* = 0.993, *p* = 0.003), and lower NEUT (*OR* = 0.719, *p* = 0.027), NLR (*OR* = 0.701, *p* = 0.022), and PLR (*OR* = 0.992, *p* = 0.016) levels. Multivariate analysis revealed that educational attainment (illiterate: *OR* = 54.247, *p* = 0.002; elementary school: *OR* = 3.126, *p* = 0.038), absence of hypertension history (*OR* = 7.306, *p* < 0.001), and UA (*OR* = 0.987, *p* = 0.002) were factors independently associated with PD-CI (compared to PS-CI). Sex (*p* = 0.071) and PLR (*p* = 0.056) showed marginal significance in the model, while age, eGFR, NEUT, and NLR were no longer significant in the multivariate model (*p* > 0.05). These findings indicate that low educational attainment, absence of hypertension history, and low UA levels are characteristics independently associated with PD-CI relative to PS-CI in this cohort, which may potentially contribute to clinical differentiation between these two types of cognitive impairment.

**Table 6:** Binary logistic regression analysis: independent correlates with group as the dependent variable.

Variable	Univariate OR (95% CI)	P value	Multivariate OR (95% CI)^1^	*P* value
Age	1.029 (0.981, 1.080)	0.236	1.016 (0.948, 1.089)	0.651
Sex (male)	0.182 (0.083, 0.398)	<0.001	0.368 (0.124, 1.090)	0.071
Education level				
Illiterate	48.167 (5.773, 401.864)	<0.001	54.247 (4.588, 641.441)	0.002
Primary school	3.513 (1.512, 8.163)	0.003	3.126 (1.066, 9.168)	0.038
History of hypertension (no)	4.030 (1.881, 8.635)	<0.001	7.306 (2.437, 21.905)	<0.001
eGFR	1.012 (1.000, 1.025)	0.054	0.992 (0.973, 1.012)	0.431
UA	0.993 (0.988, 0.997)	0.003	0.987 (0.979, 0.995)	0.002
NEUT	0.719 (0.537, 0.962)	0.027	1.010 (0.627, 1.627)	0.966
NLR	0.701 (0.517, 0.951)	0.022	1.000 (0.646, 1.548)	0.999
PLR	0.992 (0.985, 0.998)	0.016	0.987 (0.974, 1.000)	0.056

The multivariate model used the Enter method, simultaneously including age, sex, education level, eGFR, UA, NEUT, NLR, PLR, and history of hypertension. Sex with female as reference; History of hypertension with “yes” as reference; Education level with “junior high school and above” as reference. The variance inflation factor (VIF) for all variables in the model was less than 5.

### 3.7 Sensitivity analysis

To assess robustness, we repeated the ANCOVA for Hcy and UA with a reduced covariate set (age and sex only). The adjusted between-group differences remained significant for Hcy (adjusted mean difference 2.88 (95% CI 0.68–5.08) μmol/L, *p* = 0.011) and for UA (adjusted mean difference −34.58 (95% CI -64.95 to −4.21) μmol/L, *p* = 0.026), consistent with the fully adjusted model (Table 2).

## 4 Discussion

This study conducted a cross-sectional survey of 120 patients with cognitive impairment (including 60 with PD-CI and 60 with PS-CI) to systematically compare differences in Hcy and UA levels, as well as their associations with cognitive function, between the two groups. After adjusting for confounding factors such as age, sex, education level, and renal function, the study found that PD-CI patients had significantly higher Hcy levels than PS-CI patients, while their UA levels were significantly lower than those of PS-CI patients. Hcy exhibited a significant negative correlation with MMSE scores in the overall sample, with this association being more pronounced in the PD-CI group, although the intergroup difference did not reach statistical significance. No significant linear or nonlinear association was observed between UA and MMSE scores in either the overall sample or subgroup analyses. Multivariate regression analysis further confirmed that the negative impact of Hcy on MMSE scores was consistent across both groups, whereas UA did not demonstrate independent predictive value. Additionally, low educational attainment, absence of hypertension history, and low UA levels were identified as factors independently associated with PD-CI (compared to PS-CI). It is important to note that these associations do not imply causation, as the logistic regression model examined factors associated with disease type (PD-CI vs. PS-CI) rather than risk factors for developing PD-CI itself.

The above findings corroborate and extend multiple recently published studies. Regarding PD populations, Ouyang et al.’s study of 218 early-stage PD patients revealed a nonlinear relationship between Hcy and PD cognitive impairment. When Hcy levels exceeded 17.7 μmol/L, the risk of PD cognitive impairment increased significantly by 1.6-fold. This provides crucial insights into the threshold effect of Hcy-induced cognitive impairment, aligning closely with the negative correlation between Hcy and MMSE scores observed in the PD-CI group of this study (19). Another study involving 74 PD patients confirmed that serum UA levels were significantly lower in the PD-CI group compared to the cognitively intact group. Both UA and Hcy were identified as independent risk factors for cognitive impairment in PD patients, further supporting the finding of significantly reduced UA in the PD-CI group observed in this study (20). In the PS-CI domain, Hong et al.’s study of 150 patients with vascular dementia following acute cerebral infarction revealed significantly elevated UA levels in those with H-type hypertension, with MMSE scores negatively correlated with UA levels. UA was an independent risk factor for poor prognosis, suggesting that the relationship between UA and cognition may be more complex in specific subgroups. This contrasts with the lack of significant association between UA and cognition observed in the present study, which may be related to the absence of stratified analysis by hypertension subtype (21). Regarding the complex relationship between UA and cognitive function, a large-scale longitudinal study based on the UK Biobank involving over 380,000 participants with a median follow-up of 12.7 years demonstrated a U-shaped relationship between serum UA levels and Parkinsonian syndrome, and a J-shaped relationship with dementia and stroke. Furthermore, elevated UA levels were associated with reduced total brain volume and gray matter volume. Providing robust epidemiological evidence for the nonlinear role of UA in neurological disorders and explaining why a simple linear association between UA and MMSE was not observed in this study (8). Furthermore, Alrouji et al. noted that UA exhibits dual protective and detrimental effects in neurodegenerative diseases. Its neuroprotective effects primarily stem from antioxidant activity, yet prolonged hyperuricemia can lead to cognitive decline and cerebrovascular injury, further underscoring the complexity of UA’s actions (9). To date, no studies have directly compared Hcy and UA levels between patients with PD-CI and PS-CI. This study fills this gap, providing preliminary evidence for understanding the metabolic differences in cognitive impairment between these two conditions.

This study found that after adjusting for confounding factors, homocysteine levels in the PD-CI group were significantly higher than those in the PS-CI group. This difference could be speculated to be related to long-term levodopa treatment in PD patients based on previous studies. During the metabolism of levodopa by catechol-O-methyltransferase (COMT) *in vivo*, the methyl donor S-adenosylmethionine is consumed, and its metabolic byproducts directly generate Hcy. Concurrently, levodopa therapy can lead to decreased folate and vitamin B12 levels, impairing the remethylation and clearance capacity of Hcy (22). Kim et al.’s meta-analysis confirmed that PD patients receiving levodopa therapy exhibited significantly higher Hcy levels than untreated individuals, accompanied by concurrent reductions in vitamin B12 and folate levels (23). Furthermore, studies have indicated that long-term levodopa therapy without concomitant COMT inhibitor or B-vitamin supplementation results in dual alterations: elevated Hcy and decreased vitamin metabolites (24). In contrast, elevated Hcy in PS-CI patients primarily stems from nonspecific factors such as renal impairment and inadequate nutritional intake, lacking the drug-metabolism-related specific elevation mechanisms described above. This might account for the disparity in Hcy levels between the two groups, though direct evidence from the present study is lacking (6).

Furthermore, in this study, the negative correlation between Hcy and MMSE scores was numerically stronger in the PD-CI group than in the PS-CI group, although the between-group difference did not reach statistical significance. While this difference may be due to chance, the numerical trend observed in this exploratory study offers a useful hint for generating hypotheses. Therefore, under the premise of explicitly stating this as hypothesis-generating rather than confirmatory, we still explored the potential underlying mechanisms, acknowledging that this possibility remains speculative. Drawing on published literature, this trend might be attributed to Hcy’s distinct pathological pathways involved in cognitive impairment across the two diseases. In PD-CI, Hcy may directly participate in and amplify the core processes of neurodegeneration. Recent studies reveal that Hcy modifies lysine residues of target proteins through N-homocysteinylation via its metabolite homocysteinyl lactone. This process is significantly increased in the brains of PD model mice, exacerbating *α*-synuclein aggregation, mitochondrial dysfunction, and oxidative stress (25). Concurrently, NMDA receptor dysfunction—particularly excitotoxicity mediated by extrasynaptic receptors—synergizes with pathological protein aggregates like α-synuclein to drive neuronal loss (26). As an NMDA receptor agonist, Hcy may exacerbate calcium influx and excitotoxicity by overactivating extrasynaptic NMDA receptors, thereby forming a vicious cycle with PD’s intrinsic α-synuclein pathology (27). In PS-CI, however, Hcy primarily affects cognitive function indirectly through vascular injury. Elevated Hcy levels can cause endothelial dysfunction, disruption of the blood–brain barrier, and damage to cerebral microvasculature (28). Sompol et al. found in a hyperhomocysteinemic mouse model that Hcy induces astrocytic calcium signaling dysregulation, impairing neurovascular unit function including cerebral vasoregulation and synaptic regulation (29). Although these vascular mechanisms can cause cognitive decline, their effect intensity may be weaker than Hcy’s direct amplification of neurodegeneration. Therefore, Hcy may directly participate in neuronal injury and protein aggregation pathology in PD-CI, while primarily acting indirectly through vascular pathways in PS-CI. This might offer a speculative explanation for the numerically stronger correlation observed in the PD-CI group, though this remains a working hypothesis that requires validation in future studies.

This study also found that serum UA levels were significantly lower in the PD-CI group compared to the PS-CI group. Given that oxidative stress is a core pathology of PD, UA—as an endogenous antioxidant—may be extensively consumed during the process of counteracting oxidative damage, leading to decreased serum levels (9). Ren et al. further confirmed that PD patients with higher UA levels exhibited a 72% reduced risk of developing mild cognitive impairment, suggesting UA’s positive role in cognitive protection against PD (30). However, this protective effect of UA may be counteracted by the pronounced inflammatory response observed in the PS-CI group. In this study, NEUT, NLR, and PLR were significantly higher in the PS-CI group compared to the PD-CI group, indicating a more pronounced systemic inflammatory state in PS-CI patients (31). Bortolotti et al. noted that in stroke—an ischemic-reperfusion injury disease—increased xanthine oxidoreductase activity leads to excessive reactive oxygen species production, where uric acid, as a metabolic byproduct, reflects oxidative stress severity (32). Wei et al. further confirmed that patients with hyperuricemia and acute ischemic stroke exhibit significant oxidative stress damage, with elevated UA coexisting with inflammatory states (33). Thus, the higher UA levels in the PS-CI group may be associated with more pronounced inflammatory responses, potentially counteracting its neuroprotective effects. This may explain the difference in UA levels between the two groups and the complexity of its association with cognition.

This study represents the first direct comparison of Hcy and UA levels between patients with PD-CI and PS-CI within the same research framework. It systematically analyzes the association between these two biomarkers and cognitive function, as well as their heterogeneity across different disease types, providing preliminary evidence for the clinical differentiation and mechanistic understanding of these two common cognitive disorders. Concurrently, the findings offer the following clinical implications. First, elevated Hcy is a common factor associated with cognitive impairment in both PD-CI and PS-CI patients, with a consistent negative correlation with MMSE scores across groups in multivariate analysis. This suggests routine Hcy monitoring should be implemented in both patient populations, alongside exploring the feasibility of B-vitamin interventions. Second, multivariate regression analysis revealed low UA as a factor independently associated with PD-CI (relative to PS-CI). Combined with readily accessible clinical indicators such as low educational attainment and absence of hypertension history, this finding may aid in distinguishing PD-CI from PS-CI when medical history is unclear or imaging features are atypical. Third, inflammatory markers including NEUT, NLR, and PLR were significantly higher in the PS-CI group than in the PD-CI group. This suggests that cognitive impairment in PS-CI may involve more inflammatory mechanisms, whereas PD-CI is primarily driven by neurodegenerative processes. This distinction may generate hypotheses for future anti-inflammatory treatment strategies.

It must be acknowledged that this study has several limitations. First, as a single-center cross-sectional survey, the sample size was relatively limited (60 cases per group), and all participants were drawn from the same hospital, potentially introducing selection bias. This limits the possibility of conducting more in-depth subgroup analyses and restricts the generalizability of findings to broader populations. Second, the cross-sectional design can only reveal associations between variables and cannot establish causal temporal relationships between changes in Hcy or UA levels and cognitive decline. Prospective cohort studies are needed in the future to validate these findings. Third, this study did not include a healthy control group. This design choice stemmed from two considerations: First, extensive existing research has already demonstrated significant alterations in Hcy and UA levels among individuals with PD-CI and PS-CI. The core objective of this study was to compare differences in metabolic markers between these two common types of cognitive impairment and their relationship patterns with cognitive function, rather than to validate differences relative to healthy individuals. Second, enrolling healthy subjects would have required additional invasive blood collection, which was not implemented under current research conditions due to ethical considerations. Nevertheless, the absence of healthy controls limits our quantitative assessment of metabolic abnormalities between patient groups. Future studies may consider establishing matched healthy control groups to more comprehensively elucidate their clinical significance. Fourth, cognitive function assessment relied solely on the MMSE screening tool. Notably, MMSE served both as an inclusion criterion and as the sole outcome measure for cognitive performance, which may restrict the range of observed cognitive scores and potentially attenuate the true strength of associations with Hcy or UA. Given the high proportion of low-educated individuals in this region, studies indicate that the MMSE demonstrates comparable sensitivity to the MoCA in screening cognitive impairment among low-educated populations, while being simpler and more readily accepted by the elderly. However, it lacks detailed characterization of impairment in specific cognitive domains (e.g., executive function, visuospatial abilities), potentially obscuring the differential effects of Hcy or UA on distinct cognitive dimensions (34, 35). This limitation is particularly relevant given the marked educational differences between the two groups. Consequently, the conclusions drawn from this study should be interpreted as reflecting global cognitive screening performance as measured by the MMSE, rather than comprehensive cognitive function. Future studies may employ more systematic combinations of neuropsychological scales to explore the relationship between metabolic biomarkers and specific cognitive domains in greater depth. Fifth, this study conducted multiple hypothesis tests in statistical analysis but did not apply strict correction methods for multiple testing issues except for the primary analyses. The primary findings exhibit consistent effect directions and biological plausibility, with key analyses based on pre-specified research hypotheses. Furthermore, as an exploratory study, strict correction methods may increase the risk of Type II errors (false negatives), potentially obscuring findings with potential clinical significance (36), and therefore were not applied to other secondary or exploratory analyses. Nevertheless, uncorrected multiple comparisons may have elevated the risk of Type I errors (false positives) to some extent. Results should be interpreted cautiously and await confirmation in subsequent confirmatory studies. Sixth, although multiple potential confounders were adjusted for, we lacked data on other factors that could influence Hcy and UA levels, such as levodopa dosage and duration, COMT inhibitor use, vitamin B12/folate status, nutritional status, urate-lowering therapy, diuretic use, and PD severity/stage. Therefore, the mechanistic interpretations discussed above, while based on published literature, are speculative and require validation in future studies. Other unmeasured confounders may also exist. Seventh, this study assessed only baseline single-serum measurements of Hcy and UA levels, failing to reflect the cumulative effects of long-term fluctuations on cognitive function. Eighth, imaging characteristics of stroke lesions in the PS-CI group—including location, size, and number—were not analyzed. These factors may modify the association between cognitive function and Hcy/UA levels. Finally, significant differences existed between the two groups in multiple baseline characteristics. Although statistically adjusted, residual confounding cannot be entirely ruled out. Therefore, the findings of this study require further validation through larger-sample, multicenter, prospective research designs.

## 5 Conclusion

This study identified distinct Hcy and UA metabolic profiles between PD-CI and PS-CI patients: PD-CI patients exhibited higher Hcy and lower UA levels. The negative correlation between Hcy and MMSE scores was consistent across both groups in multivariate analysis, whereas no significant correlation was observed between UA and MMSE scores. Low educational attainment, absence of hypertension history, and low UA levels emerged as factors independently associated with PD-CI (relative to PS-CI), which may aid in clinical differentiation between the two forms of cognitive impairment in appropriate settings. This study provides preliminary, exploratory evidence for understanding the metabolic differences between PD-CI and PS-CI. Given the exploratory nature of this analysis, all findings should be interpreted with caution and require confirmation in future well-designed prospective studies.

## Data Availability

The raw data supporting the conclusions of this article will be made available by the authors, without undue reservation.
